# Variation in the Main Health-Promoting Compounds and Antioxidant Activity of Different Edible Parts of Purple Flowering Stalks (*Brassica campestris* var. *purpuraria*) and Green Flowering Stalks (*Brassica campestris* var. *campestris*)

**DOI:** 10.3390/plants11131664

**Published:** 2022-06-23

**Authors:** Yating Wang, Hongmei Di, Wenjuan Cheng, Guanru Ren, Sha Luo, Jie Ma, Wei Ma, Huashan Lian, Xiaomei Li, Zhi Huang, Yi Tang, Yangxia Zheng, Huanxiu Li, Fen Zhang, Bo Sun

**Affiliations:** 1 College of Horticulture, Sichuan Agricultural University, Chengdu 611130, China; 201906234@stu.sicau.edu.cn (Y.W.); 2021205035@stu.sicau.edu.cn (H.D.); 202001772@stu.sicau.edu.cn (S.L.); huangzhi@sicau.edu.cn (Z.H.); 13920@sicau.edu.cn (Y.T.); zhengyx13520@sicau.edu.cn (Y.Z.); 10650@sicau.edu.cn (H.L.); 2 Institute of Agricultural Resources and Environment, Tianjin Academy of Agricultural Sciences, Tianjin 300384, China; tjnkyxmk@tj.gov.cn; 3 College of Forestry, Sichuan Agricultural University, Chengdu 611130, China; 202101269@stu.sicau.edu.cn; 4 Bijie Institute of Agricultural Science, Bijie 551700, China; majie_011@126.com (J.M.); mw759249271@163.com (W.M.); 5 School of Agriculture and Horticulture, Chengdu Agricultural College, Chengdu 611130, China; lhs8748@163.com; 6 Rice and Sorghum Research Institute, Sichuan Academy of Agricultural Sciences, Deyang 618000, China; lxmwsl@126.com; 7 Vegetable Germplasm Innovation and Variety Improvement Key Laboratory of Sichuan, Chengdu 610300, China

**Keywords:** *Brassica campestris*, variant, edible parts, glucosinolates, antioxidants

## Abstract

Purple flowering stalks and green flowering stalks of *Brassica campestris* are widely cultivated in the middle and upper reaches of the Yangtze River. Here, concentrations of the main health-promoting compounds and antioxidant capacity levels were characterized in different parts (leaves, peel, flesh, and inflorescences) of purple and green flowering stalks. There were significant differences in the concentrations of health-promoting compounds between the two variants; the concentrations of pigments, especially anthocyanidins, and gluconapin, were significantly higher in purple flowering stalks than in green flowering stalks, and the progoitrin content was significantly higher in green flowering stalks than in purple flowering stalks. The leaves were judged to be the most nutritional edible part because they had the highest concentrations of pigments, ascorbic acid, proanthocyanidins, flavonoids, and total phenolics. Antioxidant capacity was also highest in the leaves, and it was positively correlated with the concentration of health-promoting compounds. Purple flowering stalks and green flowering stalks were found to be rich in health-promoting compounds, especially glucosinolates. Overall, our findings indicate that consumption of the leaves and peel would provide the most health benefits. Some suggestions are provided regarding the processing and utilization of these edible components.

## 1. Introduction

Two variants of *Brassica campestris* are widely cultivated in the middle and upper reaches of the Yangtze River: purple flowering stalks (*Brassica campestris* var. *purpuraria*) and green flowering stalks (*Brassica campestris* var. *campestris*). Both are popular vegetables among consumers in winter and spring in southern China.

The appearance and nutritional value of purple and green flowering stalks are major factors affecting consumer acceptance of these vegetables. The types and concentrations of pigments in vegetables have a direct effect on their appearance, which is an important indicator of the potential value of vegetables as commodities. Anthocyanidins, chlorophyll, and carotenoids are some of the major pigments in plants. Pigments provide various health benefits in addition to being used for the synthesis of color-forming substances. Anthocyanidins are non-toxic natural plant pigments that have attracted much research attention for their antioxidant functions [1]. Carotenoids, which are efficient quenchers of singlet oxygen, can scavenge free radicals and prevent the development of cancer [2]. In addition, ascorbic acid and total phenolics can provide protection against many types of cancer [2,3,4,5,6]. Proanthocyanidins can potentially be used as a health care product because of their anti-bacterial, antioxidant, anti-obesity, anti-cancer, and anti-diabetic activities [7].

Purple and green flowering stalks are cruciferous plants that are rich in important secondary metabolites, such as glucosinolates, which play an important role in plant defense and provide anticancer benefits in humans [8,9]. Approximately 200 different glucosinolates have been identified and grouped into aliphatic, aromatic, and indolic glucosinolates based on the structure of their variable side chains [10]. Glucoraphanin, glucobrassicin, and glucoiberin are the main anticarcinogenic glucosinolates [11]. Recent epidemiological studies have indicated that broccoli could function as an anti-cancer vegetable thanks to its high glucoraphanin content, reducing the risk of mammary cancer [12]. Studies with animal and cell models have suggested that the breakdown products of glucoraphanin have cancer-preventive properties, which include antioxidant, anti-inflammatory, and apoptosis-inducing activities, as well as the ability to induce cell-cycle arrest [13,14].

There are significant differences in the health-promoting compounds and antioxidant activity of the various edible parts of baby mustard (*Brassica juncea* var. *gemmifera*) [15]. In addition, the distribution and the concentrations of glucosinolates vary widely among different tissues and species of stem mustard (*Brassica juncea*) [16]. However, few studies have examined differences in the composition and concentration of health-promoting compounds between purple and green flowering stalks or in different edible parts of these plants. Here, we determined the pigments, main antioxidants, glucosinolates, and antioxidant activity in different edible parts of purple and green flowering stalks. Overall, our findings provide new information that could be used to aid the development and utilization of purple and green flowering stalks.

## 2. Results

### 2.1. Anthocyanidins, Chlorophyll, and Carotenoids

**Figure 1:**
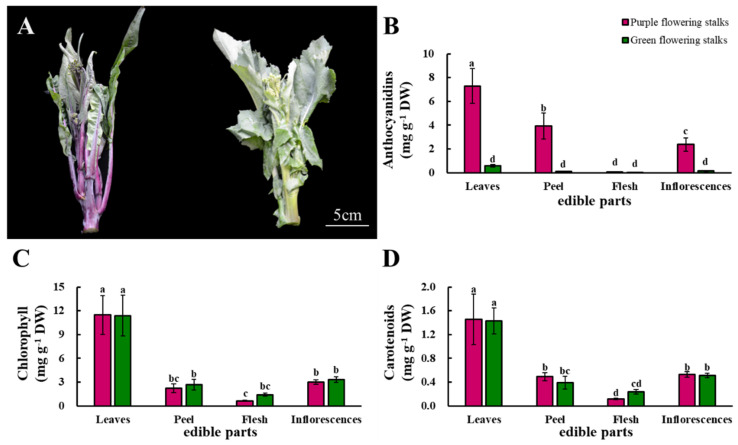
The visual appearance of purple flowering stalks and green flowering stalks (**A**) and their anthocyanidin (**B**), chlorophyll (**C**), and carotenoid (**D**) content. Same letter means no significant differences (*p* < 0.05) according to the LSD’s test.

The color difference between purple and green flowering stalks is mainly seen in the peel. The peel of purple flowering stalks is purple, whereas that of green flowering stalks is green (Figure 1A). The individual edible parts of purple flowering stalks had higher anthocyanidin content compared to those of green flowering stalks, except for the flesh. There were significant differences in the anthocyanidin content among the four edible parts of the purple flowering stalks, but the anthocyanidin content in all the edible parts of the green flowering stalks was low. The anthocyanidin content was highest in the leaves of both purple and green flowering stalks, 104.3 and 14.5 times that in the flesh, respectively (Figure 1B).

The distributions of chlorophyll and carotenoids were similar among the edible parts of the purple and green flowering stalks. There were no significant differences in the concentrations of these two substances in the same parts of the purple and green flowering stalks. The highest chlorophyll and carotenoid content in both purple and green flowering stalks was observed in the leaves, followed by the inflorescences, peel, and flesh (Figure 1C,D).

### 2.2. Ascorbic Acid

**Figure 2:**
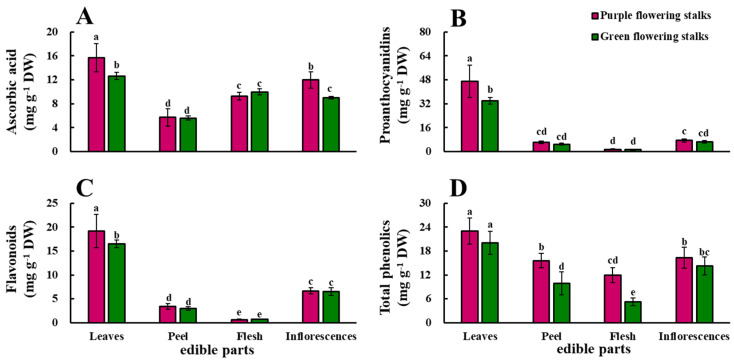
The ascorbic acid (**A**), proanthocyanidin (**B**), flavonoid (**C**), and total phenolic (**D**) content in different edible parts of the purple and green flowering stalks. Same letter means no significant differences (*p* < 0.05) according to the LSD’s test.

The ascorbic acid content was significantly higher in the leaves and inflorescences of purple flowering stalks than in the leaves and inflorescences of green flowering stalks. The ascorbic acid content significantly differed among the four edible parts of the purple flowering stalks. The ascorbic acid content was highest (15.70 mg g^−1^ DW) in the leaves and lowest (5.72 mg g^−1^ DW) in the peel. In the green flowering stalks, the ascorbic acid content was highest in the leaves (12.64 mg g^−1^ DW), followed by the flesh, inflorescences, and peel (5.58 mg g^−1^ DW) (Figure 2A).

### 2.3. Proanthocyanidins, Flavonoids, and Total Phenolics

The proanthocyanidin content was significantly higher in the leaves of the purple flowering stalks than in the leaves of the green flowering stalks. The proanthocyanidin content was low in the peel, flesh, and inflorescences of the purple and green flowering stalks, and t 30.9 and 28.4 times higher in the leaves than in the flesh of the purple and green flowering stalks, respectively (Figure 2B).

The flavonoid content was significantly higher in the leaves of the purple flowering stalks than in the leaves of the green flowering stalks. There were significant differences in the flavonoid content among the four edible parts of both the purple and green flowering stalks. The flavonoid content was highest in the leaves of the purple and green flowering stalks, followed by the inflorescences, peel, and flesh. The flavonoid content was 28.6 and 22.9 times higher in the leaves than in the flesh of the purple and green flowering stalks, respectively (Figure 2C).

The total phenolic content was significantly higher in the peel and flesh of the purple flowering stalks than in the peel and flesh of the green flowering stalks. Changes in the total phenolic content in the four edible parts of both purple and green flowering stalks were similar; the total phenolic content was highest in the leaves, followed by the inflorescences, peel, and flesh (Figure 2D).

### 2.4. Glucosinolates

**Figure 3:**
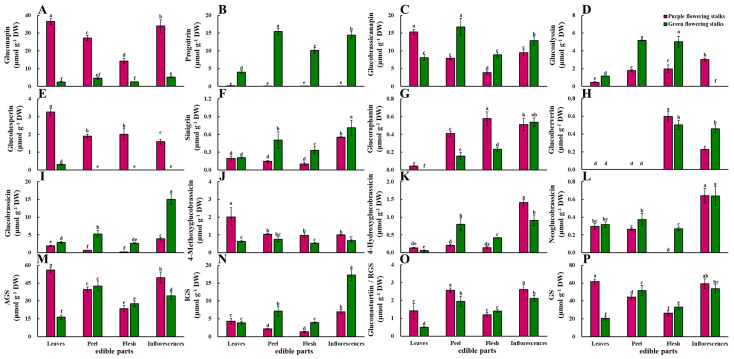
Glucosinolate content in different edible parts of purple flowering stalks and green flowering stalks. (**A**) gluconapin; (**B**) progoitrin; (**C**) glucobrassicanapin; (**D**) glucoalysin; (**E**) glucohesperin; (**F**) sinigrin; (**G**) glucoraphanin; (**H**) glucoiberverin; (**I**) glucobrassicin; (**J**) 4-methoxyglucobrassicin; (**K**) 4-hydroxyglucobrassicin; (**L**) neoglucobrassicin; (**M**) total aliphatic glucosinolates (AGS); (**N**) total indolic glucosinolates (IGS); (**O**) total aromatic glucosinolate (RGS); (**P**) glucosinolates (GS). Same letter means no significant differences (*p* < 0.05) according to the LSD’s test.

Eight aliphatic glucosinolates, four indolic glucosinolates, and one aromatic glucosinolate were detected in both purple and green flowering stalks using high-performance liquid chromatography (HPLC). The main glucosinolates in purple flowering stalks were gluconapin and glucobrassicanapin; the main glucosinolates in green flowering stalks were progoitrin and glucobrassicanapin (Figure 3).

#### 2.4.1. Aliphatic Glucosinolates

The gluconapin content was significantly higher in all parts of the purple flowering stalks relative to the corresponding parts of the green flowering stalks. The gluconapin content also significantly differed among the different parts of the purple flowering stalks. The gluconapin content was highest (36.48 μmol·g^−1^ DW) in the leaves of purple flowering stalks and lowest in the flesh (14.27 μmol·g^−1^ DW) (Figure 3A). The progoitrin content was low in the four edible parts of the purple flowering stalks, whereas there were significant differences in the progoitrin content among the four edible parts in the green flowering stalks (Figure 3B). The glucobrassicanapin content was significantly higher in the peel, flesh, and inflorescences of the green flowering stalks than in the corresponding parts of the purple flowering stalks, but the opposite pattern was observed for the leaves (Figure 3C). In purple flowering stalks, the highest glucobrassicanapin content was observed in the leaves (15.29 μmol·g^−1^ DW) and the lowest in the flesh (3.90 μmol·g^−1^ DW). In green flowering stalks, the glucobrassicanapin content was highest in the peel (16.70 μmol·g^−1^ DW) and lowest in the leaves (8.09 μmol·g^−1^ DW).

The glucoalyssin content was significantly higher in the leaves, peel, and flesh of the green flowering stalks than in the corresponding parts of the purple flowering stalks. Glucoalyssin was not detected in the inflorescences of green flowering stalks (Figure 3D). Purple flowering stalks were rich in glucohesperin, and green flowering stalks contained only a small amount of glucohesperin in the leaves (Figure 3E). The sinigrin content was significantly higher in green flowering stalks than in purple flowering stalks, with the only exception being the leaves (Figure 3F). Glucoraphanin was not detected in the leaves of green flowering stalks (Figure 3G), and glucoiberverin was not detected in the leaves and peel of both purple and green flowering stalks (Figure 3H).

The total aliphatic glucosinolates content was significantly higher in the leaves and inflorescences of purple flowering stalks than in the leaves and inflorescences of green flowering stalks. The total aliphatic glucosinolates content significantly differed among the four parts in both green and purple flowering stalks. The total aliphatic glucosinolates content was highest in the leaves of purple flowering stalks (55.89 μmol·g^−1^ DW) and the peel of green flowering stalks (42.56 μmol·g^−1^ DW) (Figure 3M).

#### 2.4.2. Indolic Glucosinolates

The glucobrassicin content was significantly higher in all parts of the green flowering stalks than in the corresponding parts of the purple flowering stalks. The glucobrassicin content was highest in the inflorescences of both the purple and green flowering stalks (3.89 μmol·g^−1^ DW and 15.04 μmol·g^−1^ DW, respectively) (Figure 3I). The 4-methoxyglucobrassicin content was significantly higher in the leaves, flesh, and inflorescences of purple flowering stalks than in the corresponding parts of green flowering stalks. The 4-methoxyglucobrassicin content was highest in the leaves (2.02 μmol·g^−1^ DW) of purple flowering stalks and in the peel (0.76 μmol·g^−1^ DW) of green flowering stalks, respectively (Figure 3J). The 4-hydroxyglucobrassicin and neoglucobrassicin contents were highest in the inflorescences of both the purple and green flowering stalks. The 4-hydroxyglucobrassicin content was significantly higher in the inflorescences of purple flowering stalks than in the inflorescences of green flowering stalks (Figure 3K). The flesh of purple flowering stalks did not contain neoglucobrassicin (Figure 3L).

The total indolic glucosinolates content was significantly higher in the peel, flesh, and inflorescences of the green flowering stalks than in the corresponding parts of the purple flowering stalks. The highest total indolic glucosinolates content was observed in the inflorescences of both the purple and green flowering stalks (6.94 μmol·g^−1^ DW and 17.27 μmol·g^−1^ DW, respectively) (Figure 3N).

#### 2.4.3. Aromatic Glucosinolate

The gluconasturtiin content, the only aromatic glucosinolate, was significantly higher in the leaves, peel, and inflorescences of purple flowering stalks than in the corresponding parts of the green flowering stalks. In the purple flowering stalks, the gluconasturtiin content was significantly higher in the peel and inflorescences than in the leaves and flesh. In green flowering stalks, the gluconasturtiin content was highest in the inflorescences and peel, followed by the flesh and leaves (Figure 3O).

#### 2.4.4. Total Glucosinolate Content

The total glucosinolate content was significantly higher in the peel and flesh of green flowering stalks than in the corresponding parts of purple flowering stalks, whereas the opposite pattern was observed in the leaves (Figure 3P).

### 2.5. Antioxidant Capacity

**Figure 4:**
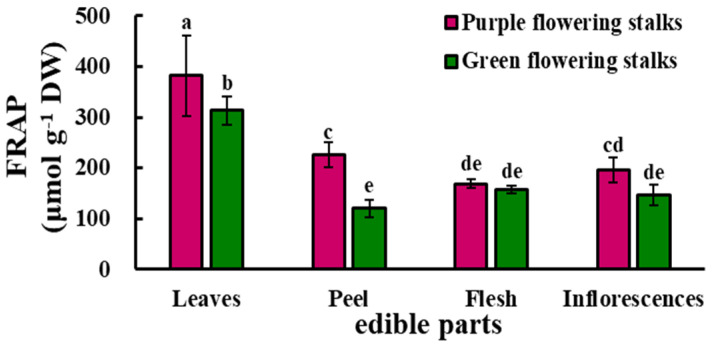
The antioxidant capacity in different edible parts of the purple and green flowering stalks. Same letter means no significant differences (*p* < 0.05) according to the LSD’s test.

The antioxidant capacity was significantly higher in the leaves and peel of the purple flowering stalks than in the corresponding parts of the green flowering stalks. The antioxidant capacity was significantly higher in the leaves than in the three other parts of both the purple and green flowering stalks, and the lowest levels were observed in the flesh (169.20 μmol·g^−1^ DW) and peel (120.69 μmol·g^−1^ DW) of purple and green flowering stalks, respectively (Figure 4).

### 2.6. PCA

**Figure 5:**
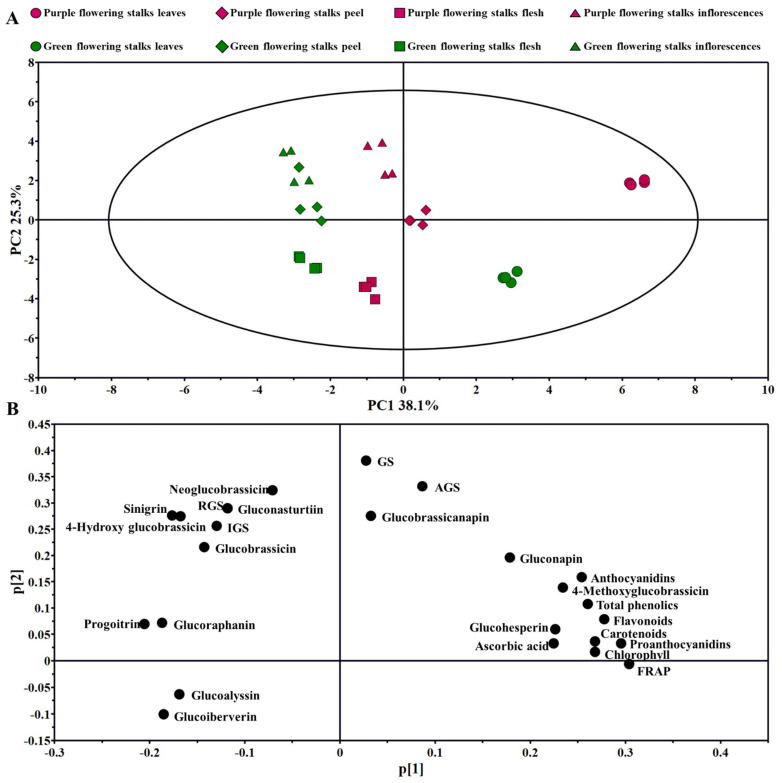
PCA analysis of different edible parts of purple and green flowering stalks. (**A**) Score plot; (**B**) loading plot. AGS: total aliphatic glucosinolates; IGS: total indolic glucosinolates; RGS: total aromatic glucosinolate; GS: total glucosinolates.

Principal component analysis (PCA) is an unsupervised multivariate analysis method. The distributions of the main health-promoting compounds and of the antioxidant capacities among the different variants and edible parts could be identified with PCA. A total of two principal components were obtained in the experiment; principal component 1 (PC1) and principal component 2 (PC2) accounted for 38.1% and 25.3% of the total variance, respectively. The leaves of purple and green flowering stalks, as well as the peel of purple flowering stalks, could be discriminated from the other parts by PC1; the flesh of purple and green flowering stalks, as well as the leaves of green flowering stalks, could be discriminated from the other parts by PC2 (Figure 5A).

According to the loading plot (Figure 5B), the major contributors to the leaves of purple flowering stalks are anthocyanidins, 4-methoxyglucobrassicin, total phenolics, flavonoids, carotenoids, and proanthocyanidins; glucoalyssin and glucoiberverin play important roles in the flesh of both purple and green flowering stalks; and sinigrin and 4-hydroxy glucobrassicin provide strong contributions to the inflorescences of green flowering stalks.

### 2.7. Correlation Analysis

**Figure 6:**
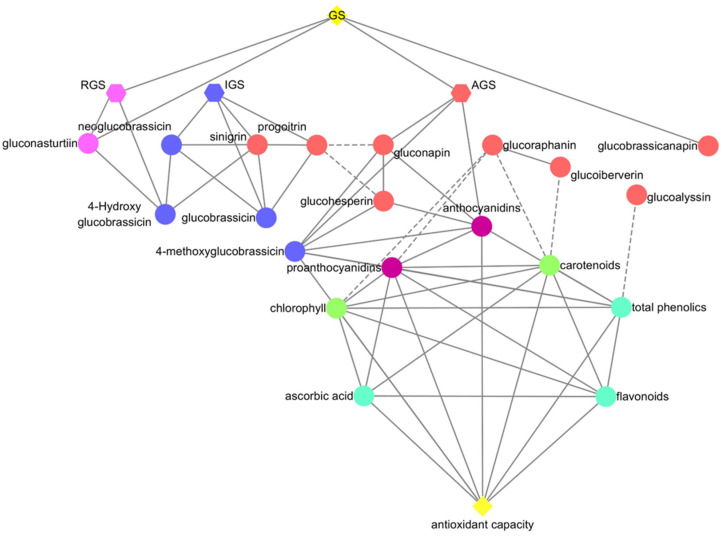
Correlation plot of the correlations between health-promoting compounds and antioxidant capacity in purple and green flowering stalks. The dashed lines between indices represent negative correlations, whereas solid lines represent positive correlations (*p* > 0.9). AGS: total aliphatic glucosinolates; IGS: total indolic glucosinolates; RGS: total aromatic glucosinolate; GS: total glucosinolates.

Total aliphatic glucosinolates, total aromatic glucosinolate, gluconasturtiin, and glucobrassicanapin were positively correlated with total glucosinolates; total aromatic glucosinolate and gluconasturtiin were positively correlated with 4-hydroxyglucobrassicin. Progoitrin was negatively correlated with gluconapin and glucohesperin. Antioxidant capacity, ascorbic acid, flavonoids, total phenolics, chlorophyll, carotenoids, and proanthocyanidins were positively correlated. Glucoalyssin was negatively correlated with total phenolics. Chlorophyll, carotenoids, and proanthocyanidins were negatively correlated with glucoraphanin, and carotenoids were negatively correlated with glucoiberverin. Total aliphatic glucosinolates, gluconapin, glucohesperin, 4-methoxyglucobrassicin, and proanthocyanidins were positively correlated with anthocyanidins (Figure 6).

### 2.8. Variance Analysis

The ratios for the edible part variance for all health-promoting compounds and antioxidant capacity were significant at the 0.01 levels, while the ratios for the variant variance for health-promoting compounds and antioxidant capacity were significant at the 0.01 or 0.05 levels, except for chlorophyll, carotenoids, and flavonoids. The ratios for the interaction (variant × edible part) variance for anthocyanidins, ascorbic acid, proanthocyanidins, and all glucosinolates were significant at the 0.01 or 0.05 levels.

The ratios for the variant variance were the highest between the variant, edible part, and interaction variances for anthocyanidins, progoitrin, gluconapin, glucohesperin, and 4-methoxyglucobrassicin.The ratios for the edible part variance were the highest for chlorophyll, carotenoids, ascorbic acid, proanthocyanidins, flavonoids, total phenolics, sinigrin, glucoraphanin, glucoiberverin, 4-hydroxy glucobrassicin, glucobrassicin, neoglucobrassicin, gluconasturtiin, total indolic glucosinolates, total aromatic glucosinolate, and antioxidant capacity. The ratios for the interaction (variant × edible part) variance were the highest for glucobrassicanapin, glucoalyssin, total aliphatic glucosinolates, and total glucosinolates (Table 1).

## 3. Discussion

The main pigments affecting the color of vegetables vary. The distinctive color of red beet and tomato stems comes from betalains and lycopene, respectively. Anthocyanidins are the main cause of the difference in color between red and green lettuce (*Lactuca sativa*) [17]. In this study, only the anthocyanidin content significantly differed in the same part between purple and green flowering stalks, indicating that the different colors of purple and green flowering stalks are mainly caused by anthocyanidins. Given that anthocyanins are not resistant to high temperature [18], the heating time for purple flowering stalks should be minimized. In addition, the peel of purple flowering stalks is not always consumed, yet the anthocyanidin content was found to be higher in the peel than in the flesh and inflorescences, suggesting that the peel could be used for the extraction of anthocyanidins in processing factories or directly consumed.

Previous studies have revealed significant differences in nutritional components among different plant organs and tissues [19,20]. In our study, the leaves contained higher levels of health-promoting compounds than other edible parts, which is useful information for the consumption and processing of different edible parts of purple and green flowering stalks. The health-promoting compound content was found to be higher in the leaves of taro (*Colocasia esculenta*) compared to other parts of the plant, especially proteins, dietary fiber, and micronutrients [21]. We found that the concentrations of three pigments, ascorbic acid, proanthocyanidins, flavonoids, and total phenolics were higher in the leaves of both purple and green flowering stalks. *Moringa oleifera* Lam leaves have received much attention in the food and medicine development fields due to their high phenolic content [22]. The leaves of purple and green flowering stalks with the highest nutrient content can not only be eaten as nutrient-dense vegetables but can also be used as a high-quality source of food coloring or for nutrient extraction.

Both purple and green flowering stalks are rich in different types of glucosinolates. Korean leaf mustard [23], tuber mustard [16], cauliflower [24], cabbage [25], and Chinese kale [26] have 8, 9, 10, 11, and 13 glucosinolates, respectively. We detected 13 glucosinolates in both purple and green flowering stalks. Aliphatic glucosinolates are a major component of the total glucosinolates in *Brassica* crops [27,28], but the predominant glucosinolates vary among species and varieties. The most abundant glucosinolate in mustard and cauliflower is sinigrin [29,30,31], whereas the most abundant glucosinolates in broccoli, Chinese kale, and loose-curd cauliflower are glucoraphanin [32], gluconapin [26], and glucoiberin [33], respectively. The most abundant aliphatic glucosinolate in purple flowering stalks was gluconapin, and progoitrin and glucobrassicanapin were the most abundant aliphatic glucosinolates in green flowering stalks.

Differences in the concentrations of the same glucosinolate in purple and green flowering stalks affect their utilization. In our study, the gluconapin content was high in purple flowering stalks and low in green flowering stalks, and the progoitrin content exhibited the opposite pattern in purple and green flowering stalks. Gluconapin is the alkenyl product of glucoraphanin [34]. Sulforaphane is a hydrolysis product of glucoraphanin and a promising anticancer agent [35]. Glucoraphanin, the precursor to gluconapin, is a more desirable compound because of its anti-cancer effect. AOP2 catalyzes the conversion of glucoraphanin to gluconapin [36]. Silencing of *BjAOP2* homologs leads to increases in glucoraphanin and glucoiberin and reductions in gluconapin, sinigrin, and glucobrassicin in *B. juncea* [37]. These findings indicate that superior purple flowering stalk varieties with higher glucoraphanin content could be created through gene editing or with silencing technology. Gluconapin is the direct precursor of progoitrin [34], and progoitrin shows goitrogenic potential in animals; however, there is no evidence that *Brassica* consumption has a goitrogenic effect in humans [38]. Therefore, purple and green flowering stalks might be useful materials for studying gluconapin and progoitrin anabolism because purple and green flowering stalks contain high levels of gluconapin and progoitrin, respectively. Furthermore, these two substances are closely related in the process of anabolism.

Antioxidant capacity reflects the comprehensive effect of various antioxidants [39]. This was confirmed by the fact that chlorophyll, carotenoids, anthocyanidins, proanthocyanidins, ascorbic acid, total phenolics, and flavonoids were all positively correlated with antioxidant capacity in our study. These substances play major roles contributing to the antioxidant capacities of different vegetables vary. Total phenolics and ascorbic acid are major contributors to the antioxidant capacity of Chinese kale sprouts [40] and baby mustard [39], respectively. We speculated that ascorbic acid and total phenolics together affect the antioxidant capacities of purple and green flowering stalks, given that changes in antioxidant capacity were similar to changes in the ascorbic acid and total phenolics contents in purple and green flowering stalks. Previous studies have shown that 4-methoxyglucobrassicin is an effective antioxidant [41], and our correlation analysis also confirmed that 4-methoxyglucobrassicin has antioxidant properties. Moreover, the antioxidant capacity of purple flowering stalks was higher than that of green flowering stalks, indicating that purple flowering stalks potentially provide more health benefits and have broader application prospects.

In recent years, with the gradual deepening of research on health-promoting compounds in horticultural plants and the rapid development of instruments and equipment, the requirements for the accuracy of high-efficiency analysis of bioactive substances are becoming more and more stringent. Precision instruments and methods are increasingly commonly used in the measurement of health-promoting compounds, such as ultra-high-performance liquid chromatography (UPLC), high-performance liquid chromatography–tandem mass spectrometry (HPLC-MS/MS), and so on [5,17,42,43,44]. In our study, we primarily chose classical spectrophotometry for content analysis, such as for pigments (anthocyanidins, carotenoids, and chlorophyll) and phenolic compounds (total phenolics, flavonoids, and proanthocyanidins), and we also hope to use more advanced methods such as this to analyze health-promoting compounds in future research and achieve accurate identification and quantification.

## 4. Materials and Methods

### 4.1. Plant Material

Purple and green flowering stalks were grown at Ya’an Experimental Base in Sichuan Agricultural University, Ya’an City, Sichuan Province. A total of 40 purple flowering stalks and 40 green flowering stalks in good condition and without disease or mechanical damage were collected for sampling and quickly transported to the laboratory. Ten purple flowering stalks and ten green flowering stalks were divided into four replicate groups for experiments. In the laboratory, the surfaces of purple and green flowering stalks were washed, dried, and separated into the leaves, peel, flesh, and inflorescences; all parts were mixed for the determination of health-promoting phytochemicals. The samples were then lyophilized in a freeze-dryer and stored at −20 °C until further analysis.

### 4.2. Anthocyanidin Content

Frozen powder was extracted with 0.1% HCl in the dark at room temperature. Then anthocyanidins content was measured by reading the absorbance at 520 nm with spectrophotometer [45].

### 4.3. Chlorophyll Content

Frozen powder (50 mg) from leaves along with other samples (300 mg) were ground and extracted with 10 mL 96.0% ethanol. After centrifugation at 4000× *g* for 5 min, the supernatant was collected and total chlorophyll content was measured by reading the absorbance at 665 nm and 649 nm with a spectrophotometer [15].

### 4.4. Carotenoid Content

Frozen powder (50 mg) was extracted with 10 mL of a mixture of acetone and petroleum ether (1:1, *v*/*v*) and petroleum ether was used to ensure constant volume. The total carotenoid content was measured by reading the absorbance at 451 nm with a spectrophotometer [15].

### 4.5. Ascorbic Acid Content

The sample powder was extracted with 1.0% oxalic acid, then centrifuged and separated at room temperature on a Waters Spherisorb C18 column (250 × 4.6 mm id; 5 μm particle size) using a solvent of 0.1% oxalic acid at a flow rate of 1.0 mL min^−1^. Each sample was filtered and analyzed by HPLC. The amount of ascorbic acid was calculated from the absorbance values at 243 nm [15].

### 4.6. Proanthocyanidin Content

Forty milligrams of sample powder was transferred to the extracting reagent, then a series of extraction operations were undertaken. Finally, the proanthocyanidin content was determined using spectrophotometry at 640 nm with a standard curve of procyanidin B2 (Sigma Chemical Co., Saint Louis, MO, USA) [46].

### 4.7. Flavonoid Content

After 24 h in the dark, the sample solution extracted with 50% ethanol was centrifuged and left for 40 min, then centrifuged at 4000× *g* for 5 min. A 1.2 mL aliquot of the supernatant was mixed with 60 µL 2% aluminum trichloride, 60 µL of 1 mol L^−1^ potassium acetate, and 1.68 mL distilled water. Absorbance was read at 415 nm using a spectrophotometer and flavonoid content was determined using a standard calibration curve [15].

### 4.8. Total Phenolic Content

The ethanol extract was mixed with Folin–Ciocalteu reagent after incubation in the dark at room temperature for 24 h, and saturated sodium carbonate was added after 3 min. The absorbance was measured at 760 nm with the spectrophotometer [15].

### 4.9. Ferric Reducing Antioxidant Power (FRAP)

The extracted samples (300 µL) were mixed with 2.7 mL of FRAP solution incubated at 37 °C. FRAP solution was prepared freshly by mixing 300 mmol L^−1^ acetate buffer (pH 3.6), 10 mmol L^−1^ 2,4,6-tripyridyl-S-triazine in 40 mmol L^−1^ HCl, and 20 mmol L^−1^ ferric chloride in a 10:1:1 *v*/*v* ratio. The absorbance was then recorded at 593 nm using a spectrophotometer after the mixture had been incubated at 37 °C for 10 min, and then the value was calculated. FeSO_4_·7H_2_O standard curves were used to calculate FRAP values [15].

### 4.10. Glucosinolate Composition and Content

Frozen powder (100 mg) was boiled in 5 mL of water for 10 min. The supernatant was collected and applied to a DEAE-Sephadex A-25 column. The glucosinolates were converted into their desulphoanalogues by treating them with aryl sulphatase, and the desulphoglucosinolates were eluted and then analyzed with an Agilent 1260 HPLC [15].

### 4.11. Statistical Analyses

All assays were performed in quadruplicate. Statistical analysis was performed using the SPSS package, version 18 (SPSS Inc., Chicago, IL, USA). Data were analyzed using two-way analysis of variance. The means were compared using the least significant differences (LSD) test at a significance level of 0.05. Principal component analysis (PCA) was performed in SIMCA-P 11.5 Demo (Umetrics, Umeå, Sweden) with unit-variance (UV) scaling to decipher the relationships among samples. The correlation results were visualized using Cytoscape v. 3.5.1 (The Cytoscape Consortium, New York, NY, USA) [39].

## 5. Conclusions

In this study, we detected major health-promoting compounds and determined the antioxidant capacity in the leaves, peel, flesh, and inflorescences of purple and green flowering stalks. Both purple and green flowering stalks are nutrient-dense vegetables, but the concentrations of their health-promoting compounds significantly differed. The anthocyanidin and gluconapin contents were high in purple flowering stalks, and the progoitrin content was high in green flowering stalks. Thus, these two vegetables could be used to study the metabolic pathways of gluconapin and progoitrin. The leaves have the highest nutritional value because they contain the most pigments and antioxidants and have the highest antioxidant capacity. Given that the anthocyanidin content in the peel of purple flowering stalks was 56.1 and 1.6 times higher than that in the flesh and inflorescences, respectively, the leaves and peel should be consumed to maximize health benefits. The leaves and peel could also be used as materials to extract nutrients.

## Figures and Tables

**Table 1:** Estimated proportions of variance components for health-promoting compounds and antioxidant capacity in purple and green flowering stalks.

Parameter	*V*_V_/*V*_P_	*V*_E_/*V*_P_	*V*_VE_/*V*_P_
Anthocyanidins	0.402 **	0.313 **	0.232 **
Chlorophyll	0.002	0.925 **	0.001
Carotenoids	0.000	0.902 **	0.006
Ascorbic acid	0.042 **	0.806 **	0.062 **
Proanthocyanidins	0.013 *	0.917 **	0.026 *
Flavonoids	0.004	0.959 **	0.007
Total phenolics	0.148 **	0.685 **	0.029
Gluconapin	0.787 **	0.111 **	0.092 **
Progoitrin	0.740 **	0.126 **	0.127 **
Glucobrassicanapin	0.091 **	0.328 **	0.521 **
Glucoalyssin	0.079 **	0.420 **	0.483 **
Glucohesperin	0.830 **	0.108 **	0.048 **
Sinigrin	0.190 **	0.642 **	0.083 **
Glucoraphanin	0.123 **	0.728 **	0.119 **
Glucoiberverin	0.005 **	0.921 **	0.062 **
Glucobrassicin	0.289 **	0.504 **	0.191 **
4-Methoxyglucobrassicin	0.413 **	0.221 **	0.228 **
4-Hydroxy glucobrassicin	0.006 *	0.768 **	0.196 **
Neoglucobrassicin	0.060 **	0.811 **	0.070 **
Total aliphatic glucosinolates	0.218 **	0.258 **	0.480 **
Total indolic glucosinolates	0.210 **	0.595 **	0.173 **
Gluconasturtiin	0.106 **	0.735 **	0.084 **
Total aromatic glucosinolate	0.106 **	0.735 **	0.084 **
Total glucosinolates	0.073 **	0.437 **	0.438 **
Antioxidant capacity	0.107 **	0.750 **	0.036

*V*_V_/*V*_P_: ratio of variant variance to phenotypic variance; *V*_E_/*V*_P_: ratio of edible part variance to phenotypic variance; *V*_VE_/*V*_P_: ratio of variant × edible part interaction variance to phenotypic variance. * and ** indicate the significance at 0.05 and 0.01 probability levels in the same column, respectively.

## Data Availability

The data presented in this study are available in the manuscript and Supplementary Materials.

## Supplementary Materials

The following supporting information can be downloaded at: https://www.mdpi.com/article/10.3390/plants11131664/s1, Table S1: Correlation coefficients between health-promoting compounds and antioxidant capacity in purple and green flowering stalks.
